# Giant Cell Tumor of Tendon Sheath of the Distal Phalanx

**DOI:** 10.7759/cureus.29461

**Published:** 2022-09-22

**Authors:** Ahmad N Boeisa, Ali A Al Khalaf

**Affiliations:** 1 Pediatric Orthopedic Surgery, Almoosa Specialist Hospital, Al-Ahsa, SAU; 2 College of Medicine, King Faisal University, Hofuf, SAU

**Keywords:** index finger, soft-tissue tumor, distal phalanx, tendon sheath, tenosynovial giant cell tumor

## Abstract

Giant cell tumor of tendon sheath (GCTTS) is a benign proliferative disorder of the synovial joints and tendon sheaths that typically manifests as a painless, firm, localized, and slow-growing mass. Commonly seen among women in the third to fifth decades, GCTTS can be diagnosed clinically; however, histopathological confirmation is required. The tumor is primarily removed surgically. Recurrence after excision is possible and occurs in up to 45% of cases.

## Introduction

A tenosynovial giant cell tumor of tendon sheath (GCTTS) is a benign proliferative disorder of the synovial joints and tendon sheaths that typically manifests as a painless, firm, localized, slow-growing mass [1]. A GCTTS can be localized or diffuse, with nodular or multilobular growth, which can impair tendon and joint movement [2]. It can happen at any age, but women in their third to fifth decades have the highest incidence [3]. Histopathological examination of the tumor in conjunction with clinical and radiographic findings is used to make the diagnosis [4]. The most effective treatment for GCTTS is complete surgical excision [3]. The risk of recurrence is considerably high, reaching up to 30-45% [5]. Long-term follow-up after excision is advised in order to detect early recurrence.

## Case presentation

**Figure 1:**
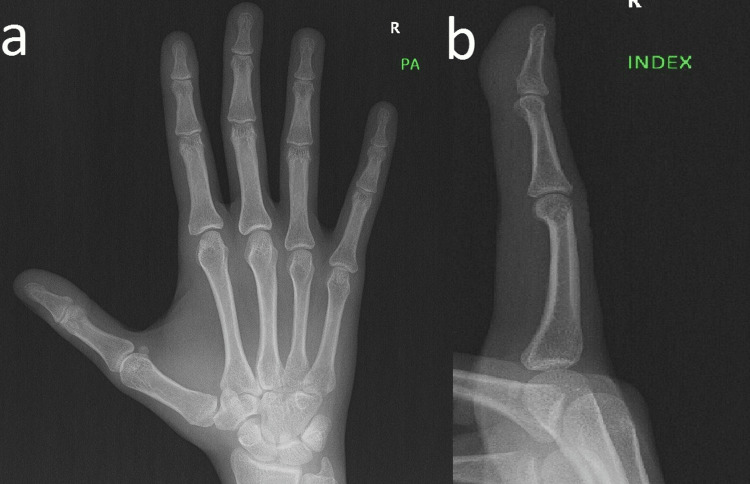
Posteroanterior X-ray of the right hand (a) and lateral view of the right index finger (b) Showing normal bones (no radiographic abnormalities) and soft tissue swelling in the bulb of the index

A 30-year-old female, medically free, presented to our clinic with localized, painless swelling of the distal phalanx of the right index finger, which was noticed one year ago. The swelling was progressively getting bigger over a period of one year. No history of trauma was noted. On examination, 1×1 cm firm soft tissue mass from the volar bulb of the right index finger, mobile, and attached to a tendon was noted. The full range of motion of the distal interphalangeal joint could be obtained. Neurological examination revealed no abnormalities. A differential diagnosis of giant cell tumor and ganglionic cyst were considered based on history and clinical examination. X-ray of the right hand and the right index finger revealed no fracture, dislocation, or destructive lesion (Figure 1a and Figure 1b).

**Figure 2:**
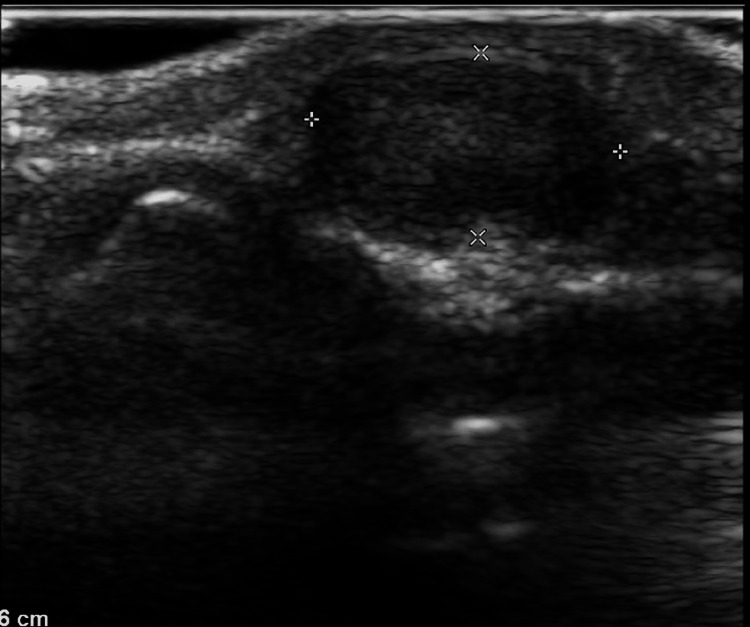
Ultrasound examination of the tumor Ultrasound examination revealed a 9×5 mm cyst, with turbid content, adjacent to the distal phalanx with no internal perfusion or affection of the underlying bone. There was no evidence of increased surrounding vascularity.

An ultrasound examination revealed a 9×5 mm cyst, with turbid content, adjacent to the distal phalanx, with no internal perfusion or affection of the underlying bone. There was no evidence of increased surrounding vascularity (Figure 2).

**Figure 3:**
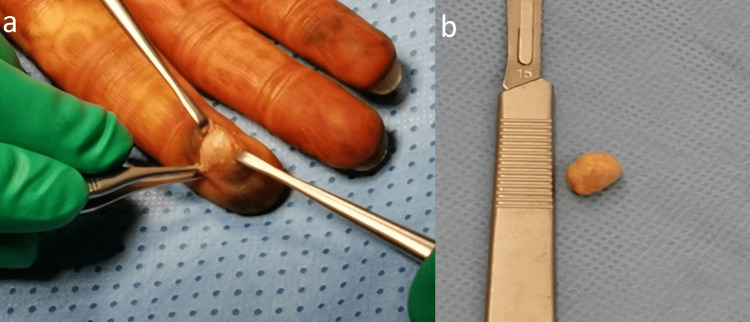
Tumor resection intraoperative (a) and after complete resection (b) Complete resection of the tumor

Complete surgical excision was performed under local anesthesia. An oval-shaped, tan-yellow mass measuring 1×0.9×0.5 cm was completely removed (Figure 3a and Figure 3b). Suspicion of a giant cell tumor was raised, indicating a histopathological confirmation.

A histopathological examination showed a lobulated and well-defined tumor with a fibrous pseudocapsule, composed of mononuclear cells with eosinophilic cytoplasm and vesicular nuclei, Xanthomatous cells, siderophages, osteoclast-like multinucleated giant cells, and mononuclear inflammatory cells. The cells were surrounded by a prominent collagenous stroma that had varying degrees of hyalinization. The diagnosis of a tenosynovial GCTTS was confirmed. A follow-up plan was established for the patient after two and six weeks and then every six months. After two months of tumor excision, the patient returned for two follow-up visits with no complaints and no signs of recurrence.

## Discussion

Giant cell tumor of tendon sheath (GCTTS) is a benign tumor that can be locally aggressive [1,2]. Found most commonly in the epiphysis and metaphysis of long bones [6], it is the second most common type of tumor seen in the hands [7,8]. According to published cases, giant cell tumors most commonly affect the first three fingers, with the index finger being the most frequently involved [8], and are most commonly found in the region of the distal interphalangeal (DIP) joint, followed by the proximal phalanx [3,7,9]. GCTTS is most common in women in their third to fifth decades, but it can occur at any age or gender [5].

Clinical diagnosis is possible, but histopathological examination is required to confirm the diagnosis [4]. GCTTS manifests clinically as a firm, painless, slowly growing focal mass that can impair the mechanical function of the involved tendon and joint [1,2]. Soft tissue swelling is commonly seen on radiographs [3,10]. X-rays are also used to determine whether a fracture, dislocation, or destructive lesion exists. Sonography can be used to distinguish between solid and cystic lesions and to detect satellite lesions. Furthermore, it can demonstrate the tumor's relationship with the surrounding structures. Sonography can provide information on the extent of contact with the underlying tendon as well as the percentage of circumferential involvement [10]. MRI is the preferred imaging modality, particularly for evaluating extra-articular manifestations [3]. It is a valuable modality for the preoperative diagnosis of GCTTS when imaging findings are carefully analyzed in conjunction with clinical information, especially if core features are detectable [11].

In our case, based on the history and clinical examination, we suspected GCTTS and ganglion cysts. An ultrasound revealed that a ganglion cyst was more likely than a giant cell tumor. However, after surgical excision, the tumor's characteristics were more suggestive of a giant cell tumor, indicating histopathological confirmation. A histopathological examination was performed, which confirmed the diagnosis of GCTTS. This emphasizes the significance of employing a high-resolution imaging modality in the evaluation of such unclear lesions.

A histopathological examination of the excised tissue confirms the diagnosis [4]. Histopathologically, GCTTS is composed of a variety of cells, including multinucleated giant cells, polyhedral histiocytes, fibrotic material, and hemosiderin deposits [12-14]. Complete surgical excision is the primary treatment for GCTTS [3]. Because of the presence of a pseudocapsule, the tumor is frequently removed as a whole [3]. To prevent recurrences, satellite lesions and connections should be excised completely. Given the reported high recurrence rate of up to 45%, long-term follow-up after excision is advised [5].

Several characteristics of GCTTS have been linked to an increased risk of recurrence, including tumor size, nodularity, mitotic rate, and cellularity [15,16]. The location of the tumor has been linked to the rate of recurrence [15,17,18]. The recurrence of giant cell tumors was significantly higher at the thumb interphalangeal (IP) and distal digital interphalangeal (DIP) joints according to Reilly et al. [15,17,18]. It could be argued that excising the tumor distally at the proximal interphalangeal (PIP) joint or the thumb IP and DIP joint levels is difficult due to the proximity of neurovascular structures to tumor margins [7,15].

## Conclusions

In our case, based on the history and clinical examination, we suspected GCTTS and ganglion cysts. An ultrasound report revealed that a ganglion cyst was more likely than a giant cell tumor. However, after surgical excision, the tumor's characteristics were more suggestive of a giant cell tumor, indicating histopathological confirmation. A histopathological examination was performed, which confirmed the diagnosis of GCTTS. This emphasizes the significance of employing a high-resolution imaging modality in the evaluation of such an unclear diagnosis.
